# Crystallinity and β Phase Fraction of PVDF in Biaxially Stretched PVDF/PMMA Films

**DOI:** 10.3390/polym13070998

**Published:** 2021-03-24

**Authors:** Ye Zhou, Wenting Liu, Bin Tan, Cheng Zhu, Yaru Ni, Liang Fang, Chunhua Lu, Zhongzi Xu

**Affiliations:** 1 State Key Laboratory of Materials-Oriented Chemical Engineering, College of Materials Science and Engineering, Nanjing Tech University, Nanjing 210009, China; zhouye3279@163.com (Y.Z.); 201861103065@njtech.edu.cn (W.L.); chhlu@njtech.edu.cn (C.L.); zzxu@njtech.edu.cn (Z.X.); 2 Jiangsu Collaborative Innovation Center for Advanced Inorganic Function Composites, Nanjing Tech University, Nanjing 210009, China; 3 Jiangsu National Synergetic Innovation Center for Advanced Materials (SICAM), Nanjing Tech University, Nanjing 210009, China; 4 Department of Chemical Engineering and Materials Science, Michigan State University, East Lansing, MI 48824, USA; tanbin1@egr.msu.edu

**Keywords:** PVDF, PMMA, thermo-mechanical fields, biaxial stretching, β phase, crystallinity

## Abstract

Polyvinylidene fluoride (PVDF) and poly(methyl methacrylate) (PMMA) blend films were prepared using biaxial stretching. The effects of PMMA content and stretching ratio on the crystallinity and β phase fraction of PVDF in blend films were investigated. The distributions of crystallinity and β phase fraction on variable locations were also studied. The results of FTIR and XRD showed that β phase appeared in PVDF/PMMA blends after extrusion and casting procedures. Although β phase fraction decreased after preheating, there was still an increasing trend during following biaxial stretching. More importantly, the increase in PMMA content improved β phase fraction, and the highest β phase fraction of 93% was achieved at PMMA content of 30 wt% and stretching ratio of 2×2. Besides, the reduction in PMMA content and the increase in stretching ratio improved the crystallinity of PVDF. The mechanical properties of the stretched films were significantly improved by increasing the stretching ratio as well. The uniform stress distribution on different regions of biaxial stretching films contributed to the uniform distribution of β phase fraction and crystallinity of PVDF with the aid of simulation. This work confirmed that biaxial stretching can be a candidate method to prepare PVDF/PMMA blend films with uniform distributions of comparable β phase and crystallinity of PVDF.

## 1. Introduction

Polyvinylidene fluoride (PVDF) as one of piezoelectric polymers is widely used in flexible sensors, actuators, and self-powering devices [1,2,3,4], etc. Many unique properties of PVDF and its copolymers are contributed by electrical dipole moments of VDF monomers, resulting from the electronegativity of fluorine atoms [5,6]. Semi-crystalline PVDF shows mainly five crystalline phases, including α and β phases. Due to all trans planar zigzag chain conformation, β phase shows the highest dipolar moment per unit cell [7]. In comparison, α phase is non-polar because of antiparallel packing of dipoles [5,8]. Hence, there is a need to enhance β phase in PVDF specimens to improve their performance in desirable applications [9].

Blending is a common method to improve β phase in PVDF specimens. It has been well known that the addition of poly(methyl methacrylate) (PMMA) affects PVDF crystallization kinetics and more importantly alters its crystal phases [10,11,12,13]. The interaction between oxygen atoms of carbonyl groups in PMMA and hydrogen atoms in PVDF plays a critical role [10,14,15,16,17]. Composite fillers within PVDF matrix is also considered as another option to improve crystallinity of PVDF matrix due to the reduction in free energy barrier for nucleation [18]. The interaction between negatively charged particles and positive charged CH_2_ groups of PVDF contributes to the improvement in β phase [19,20,21]. BaTiO_3_ as an excellent piezoelectric ceramic material was used to nucleate electroactive β phase with a maximum β phase of 82% [22]. Another report shows that the β phase fraction of 99% was obtained for the phosphonium surfactant modified clay [23].

Preparation methods are critical to alter β phase in PVDF specimens. So far, the evolution of crystalline phase transition of PVDF has been widely studied. Many processing methods were used to prepare PVDF samples with different fraction of β phase. Solvent processing is one of the most common methods such as spin coating, solution casting, Langmuir Blodgett deposition process, electrospinning and so on [24,25,26,27]. Solvent polarity can affect the formation of β phase. A solvent with high dipole moment facilitates dipole alignment of polymer chains. Nearly pure β phase was achieved when DMSO was used [25]. During Langmuir Blodgett deposition, β phase of PVDF in ultra-thin films can be controlled parallel to the substrates and the dipoles were aligned perpendicular to the substrates. Highly pure β phase, thus, was achieved [26]. The study on the interaction between polar solvent and PVDF can facilitate the understanding of β phase formation in VM films where the interaction between the CH_2_ groups in PVDF and the carbonyl (C=O) groups in PMMA serves as the key role to induce β phase. The steady shear stress was introduced to PVDF during spin-coating that can work as a mechanical stretching. The speed of spin coating and the followed baking temperature are two important factors to control shearing force and crystallization time. A slow evaporation of the solvent and in turn relatively slow crystallization induced the formation of a high amount of β phase (94%) [24]. Spin coating can produce a thin film with thickness from nano-scale to micro-scale. The film thickness can be well controlled by spin speed and solution concentration. The thickness of spin coating film can be reduced effectively by increasing spin coating speed or decreasing solution concentration [28,29,30]. Besides, forcespinning and electrospinning were used to create PVDF fibers. It was found that the electric field was more important than mechanical field in spinning to induce preferred dipole orientation in electrospun PVDF fibers due to in situ poling effect [27].

Because solvent processing cannot be used easily in industry, thermo-mechanical processinng is another main method to induce β phase of PVDF. The role of thermo-mechanical fields that are determined by melt processing methods, thus, should not be overlooked, including pressure-quenching, pressing-and-folding, non-isothermal crystallization, extrusion-rolling, uniaxially stretching and so on [31,32,33,34,35]. Cooling rate is a critical factor, and the β phase can be induced by quenching. When the cooling rate was increased above 2000 K·s^−1^, pure β phase was mainly formed in PVDF [33]. Compression using high pressure can also create high amounts of β phase. For example, β phase fraction can reach 95% at a high pressure of 640 MPa. Besides, pressing-and-folding cycles were carried out with the pressure of 300 kN to achieve 98% β phase [32]. When stretching was concerned, to date, most investigations focused on rolling or uniaxially stretching that are available for industrial production. 98% β phase was achieved by extrusion and rolling [35]. High and low shear mixers were used to create PVDF/PMMA blends with the mass ratio of 80/20 and 60/40. Independent of processing conditions, the fraction of β phase of PVDF was increased via the addition of PMMA, i.e., the fraction was increased from 50% to 54% and 64% for PVDF/PMMA = 80/20 and 60/40 in a low shear mixer (100 rpm), respectively. The fusion temperature (Tf) and degree of crystallinity (Xc) were decreased by increasing shearing speed and PMMA amount [33].

Post-stretching offers a further opportunity to induce orientation of polymer chains in thin films, which facilitates the achievement of required structures, properties and functions [5,36,37,38]. Uniaxial stretching along machine direction (MD) is a basic post-stretching mode in polymer film industry. The structures and properties are different along MD and transverse direction (TD). It was reported that the uniaxial stretching of PVDF films to 500% at 90 °C created β phase of 74% due to re-alignment of polymer chains and all-trans planar conformation [5]. Mechanical stretching is also a common method to tune crystalline structures and properties of PVDF blends. For example, non-polar *α* phase in the blend of PVDF copolymers was transformed into β phase after stretching, and β phase fraction was increased from 55% to 67% [39]. 

In comparison with uniaxial stretching, biaxially stretched polymer films can find great applications because of well-controlled film thickness and isotropic properties along MD and TD [34,40]. Biaxially oriented PVDF films were achieved with stretching ratios of 2 × 2, 2.5 × 2.5, 3 × 3, and 3.5 × 3.5 at 170 °C. Both crystallinity and β phase content as well as tensile strength were increased by increasing stretching ratio [41]. The effects of temperature and stretching ratio were studied in another research. A low temperature and a high stretching ratio increased the crystallinity and the fraction of β phase, both of which are lower than that prepared by uniaxial stretching at the same temperature [34]. However, there is few reports on the β phase fraction and crystallization of PVDF in biaxially stretched PVDF blends films. The uniformity of crystallinity and β phase in different areas of biaxially stretched films has not been investigated well, which dominates the quality stability of PVDF based products. 

In the present work, we investigated the crystallinity and β phase fraction of PVDF in biaxially stretched PVDF/PMMA films. We first prepared PVDF/PMMA blends sheets through extrusion and casting stages. The mass ratio of PVDF/PMMA blends was controlled as 85/15, 80/20, 75/25, and 70/30, while the stretching ratio was 2 × 2, 2.5 × 2.5, 3 × 3, respectively. The effects of mass ratio and stretching ratio on crystallinity and β phase fraction of PVDF were studied. Their distributions on different regions of the stretched films were also studied via experiments and simulations.

## 2. Experimental Section

### 2.1. Materials

Polyvinylidene fluoride (PVDF, DS206, Huaxia Shenzhou Co Ltd., Zibo, China), polymethyl methacrylate (PMMA, CM205, Qimei Co Ltd., Zhenjiang, China), dimethicone (AK1000, Wacker Co Ltd., Munich, Germany) were used without purification. The material parameters of PVDF and PMMA have been added in Table S1.

### 2.2. Preparation of PVDF/PMMA Pellets

**Figure 1:**
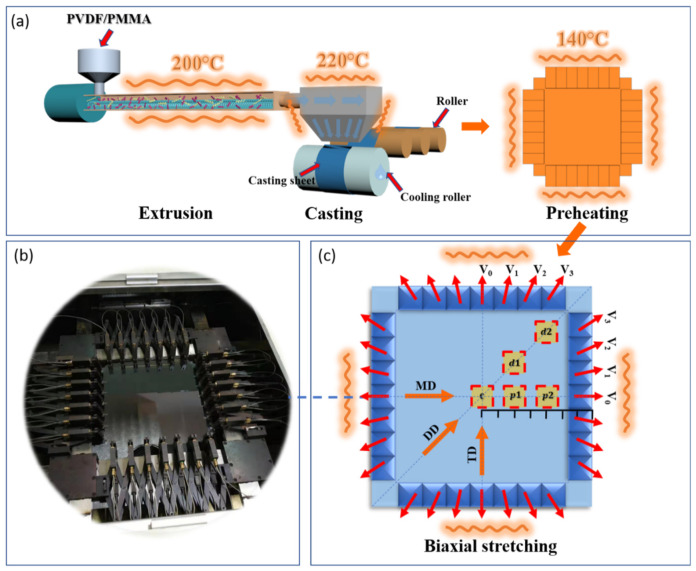
(**a**) Schematic diagram of extrusion, casting, preheating and biaxial stretching processing. (**b**) Photo of the biaxially stretching device. (**c**) Schematic diagram of the film during stretching.

As shown in Figure 1, PVDF and PMMA particles with different mass ratios (85/15, 80/20, 75/25 and 70/30) were stirred for 20 min in a blender (LSH-800, Baixiong Co Ltd., Zhangjiagang, China) with the aid of dimethicone. Then, the blends were prepared by twin-screw extrusion (EC-52, Yuesheng Co Ltd., Nanjing, China) at 200 °C. The die temperature was 220 °C and a high-speed shearing pelletizer (LAB-30, Yuesheng Co Ltd., Nanjing, China) was used to prepare pellets. 

### 2.3. Preparation of PVDF/PMMA Casting Sheets

Casting blends sheets were prepared using rotating rolls that were cooled by water circulation as shown in Figure 1. The thickness of these sheets was controlled as 350–400 μm by changing the speed of rotating rollers and the width of the die mouth. The dimensions of the die, the cooling roll and the rotation speeds during the casting have been shown in Figure S1.

### 2.4. Biaxial Stretching of PVDF/PMMA Films

Casting blends sheets with the dimension of 100 × 100 × 0.4 mm^3^ were biaxially stretched with different stretching ratios by a two-direction planar stretching machine (LHLS08, Lihua Co Ltd., Nanjing, China). Before stretching process, blends sheets preheated in the machine at 140 °C for 40 s, which were then stretched constantly at a speed of 3.6 mm s^−1^ to desired stretching ratios, as shown in Figure 1.

As shown in Table 1, the PVDF/PMMA blend (VM) films with different PVDF/PMMA mass ratios of 85/15, 80/20, 75/25 and 70/30 were successfully biaxially stretched to different stretching ratios. When PMMA mass ratio was less than 15 wt %, the PVDF/PMMA sheets were not stretched successively. The VM films were biaxially stretched to the ratio of *R* = 2 × 2, 2.5 × 2.5, and 3 × 3. Table 2 shows the thickness of biaxial stretch films, which was strongly influenced by stretching ratios. When the film thickness was less than 20 μm, the stretch fracture often occurred.

### 2.5. Characterizations

Differential scanning calorimetry (DSC, TA Instruments, Newcastle, DE, USA) was used to characterize the melting and crystallization peaks of PVDF and a PVDF/PMMA blend. The sample was heated from room temperature to 190 °C at the rate of 10 °C min^−1^ before it was cooled to 60 °C at the same rate. The cooling curve and the second heating curves were reported.

We also investigated the β phase fractions and crystallinities of PVDF in PVDF/PMMA blends during processing by Fourier transform infrared spectroscopy (FTIR, Nicolet, Madison, WI, USA) and X-ray diffraction (XRD, Rigaku, The Woodlands, TX, USA). X-ray diffraction (XRD) measurements of prepared samples were conducted on an X-ray diffractometer (SmartLab-3KW, Rigaku) employing Cu Kα radiation (λ = 0.154059 nm) at a scanning rate of 10 °/min over the range of 10° ≤ 2θ ≤ 80°. Fourier transform infrared spectroscopy (FTIR) was conducted on a V PerkinElmer frontier infrared spectrometer in the spectral range of 4000–400 cm^−1^, with resolution of 4 cm^−1^.

In addition, mechanical properties were measured using a C610M tensile testing machine (Labthink, Jinan, China) at room temperature with a crosshead speed of 50 mm min^−1^ according to GB/T 1040.2-2006. 

To study the stress distributions on films during biaxial stretching, finite element method (FEM) simulations were carried out using COMSOL. 

## 3. Result and Discussions

### 3.1. β Phase Fractions and Crystallinities of PVDF during Extrusion and Casting

**Figure 2:**
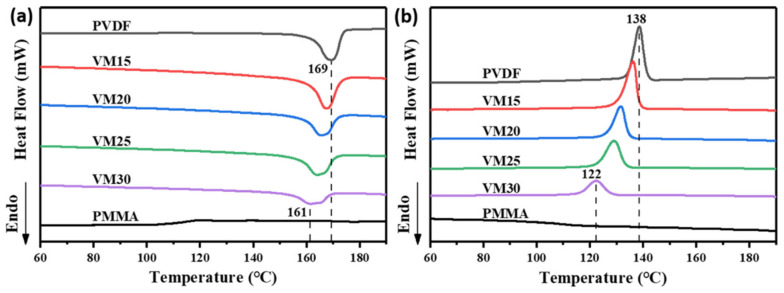
DSC (**a**) heating and (**b**) cooling thermograms of PVDF, PVDF/PMMA blend VM and PMMA.

The melting and crystallization temperatures of PVDF, PVDF/PMMA blends VM and PMMA were first measured to determine suitable processing windows. As shown in Figure 2, because of amorphous structure, none melting and crystallization peak appeared in the PMMA DSC curve. Figure 2a shows the second heating curves, the pristine PVDF exhibited a melting peak at 169 °C. With the addition of PMMA, the melting peaks of VM were obviously decreased to 161 °C. In Figure 2b, the crystallization peaks also decreased with the addition of PMMA, from 138 °C of the pristine PVDF to 122 °C.

As shown in Figure 1, VM pellets were subjected to high-speed shearing during the extrusion before the casting stage. The casting sheets were then preheated at 140 °C and went through biaxial stretching. The schematic diagram of the film during stretching was shown by Figure 1c, the small blue squares represent 28 fixtures, whose moving directions are represented by the red arrows. The big azure square represents the film, the yellow squares are five different (c, p1, p2, d1, d2) locations on the film, and the orange arrows represent directions of machine direction (MD), diagonal direction (DD) and transverse direction (TD) in the film. Because the crystalline behaviors of VM can be highly affected by extrusion, casting and preheating processing, it is necessary to study the β phase fraction and crystallinity of PVDF during these primary stages.

**Figure 3:**
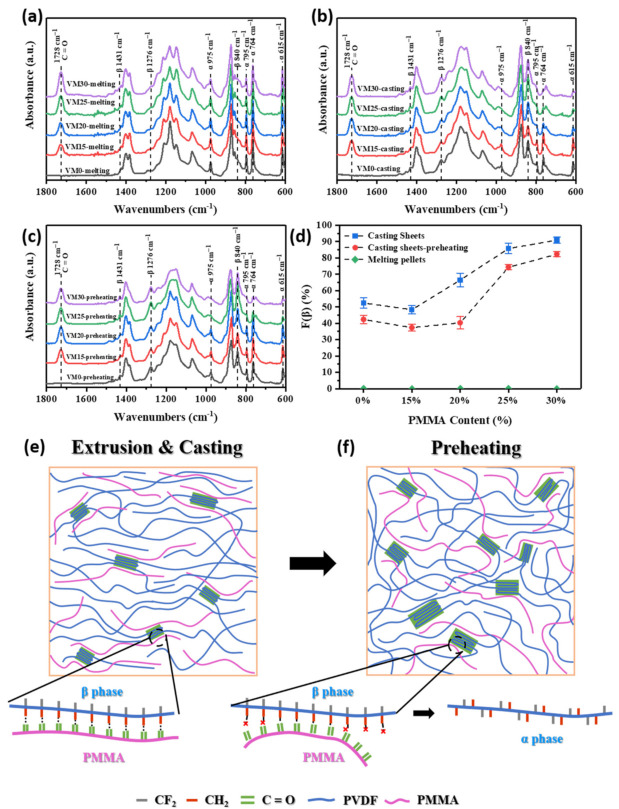
FTIR spectra of (**a**) the thermal-history free VM; (**b**) VM casting sheets; (**c**) VM casting Scheme 140 °C; and their (**d**) β phase fractions. (**e**,**f**) Schemes of chain orientations and evolution of α-β phases in casting sheets during extrusion, casting and preheating procedures.

VM suffered high-shearing during the extrusion and following quenching process. Therefore, we heated VM pellets at 220 °C for 10 min and allowed them to cool down to room temperature in atmosphere to remove thermal history. The FTIR spectra of the thermal-history free blends are shown in Figure 3a. Obvious characteristic peaks of α phase were observed at 615, 764, 795, and 975 cm^−1^. Neglected peaks of β phase were found, indicating that the content of β phase can be ignored. As far as the casting sheets that went through both extrusion and casting stages were concerned, peaks of β phase at 840, 1276 and 1431 cm^−1^ were noted (Figure 3b), indicating the appearance of β phase [34,40,42,43]. The presence of the characteristic peaks at 1728 cm^−1^, attributable to the stretching of the carbonyl group (C=O), was used to characterize the existence of PMMA in the blend [14,44]. As shown in Figure 3c, when the casting sheets were preheated at 140 °C for 40 s, which is the same condition before biaxial stretching, the peak at 764 cm^−1^ assigned to α phase was enhanced relative to the peak at 840 cm^−1^ assigned to β phase.

To quantitively investigate the fraction of *β* phase (F(*β*)), the following equation was used:

(1) $$F\left(\beta \right)=\;\frac{A_{\beta }}{\left(\frac{K_{\beta }}{K_{\alpha }}\right)A_{\alpha }+A_{\beta }}=\;\frac{A_{\beta }}{\left(1.26\right)A_{\alpha }+A_{\beta }}$$

where, the values of *A_α_* and *A_β_* represent the absorbance of the peaks at 764 and 840 cm^−1^, respectively. *K_α_* = 6.1 × 10^4^ cm^2^ mol^−1^ and *K_β_* = 7.7 × 10^4^ cm^2^ mol^−1^ are absorption coefficients. As shown in Figure 3d, F(*β*) was almost close to 0 when the extruded pellets were heated again and cooled down in ambient atmosphere. Relatively high F(*β*) was obtained in the casting sheets, which was decreased after the preheating procedure. The preheating temperature of 140 °C that is close to the crystallization temperature of PVDF facilitated the formation of thermodynamically stable α phase crystals. When the PMMA content was 30 wt%, the β PVDF fraction reached nearly 90%.

The evolution mechanism of β phase during casting and preheating procedures are shown in Figure 3e,f. The casting procedure led to the uniaxial stretching of the sheet, which is beneficial to the formation of β phase. Besides, the interaction between oxygen atoms of C=O groups in PMMA and hydrogen atoms of CH_2_ groups in PVDF induced the formation of β crystals [17]. During preheating, the oriented molecular chains especially PMMA relaxed at a high temperature, which weakened the interaction between C=O groups and CH_2_ groups, reducing the β phase fraction.

**Figure 4:**
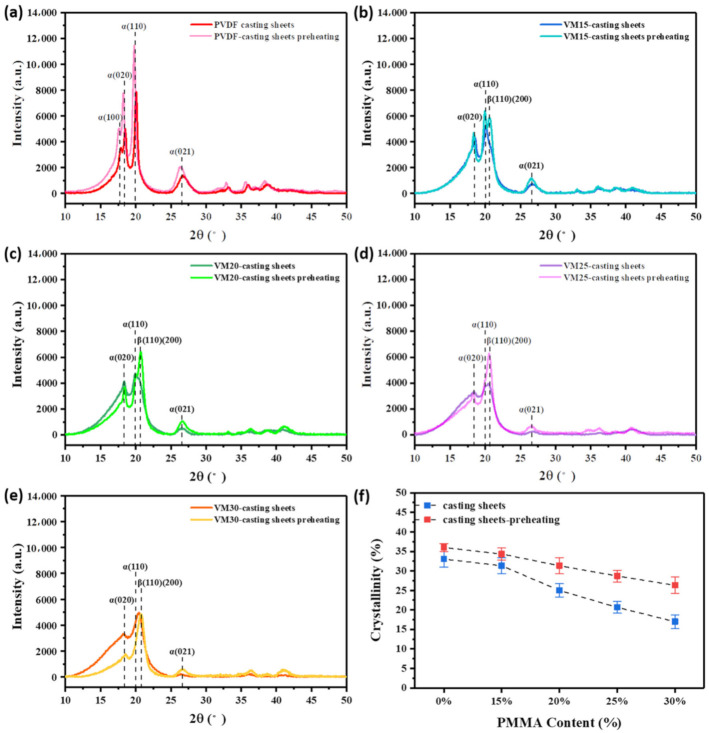
(**a**–**e**) XRD patterns of VM casting films before and after preheating; (**f**) Crystallinities of VM casting films before and after preheating.

In addition to β phase fraction, total crystallinity is critical as well for PVDF films [25]. XRD analysis was performed to study the crystallization of VM in these primary stages. Figure 4 shows the diffraction peaks at 2*θ* = 17.8°, 18.4°, 19.9°, which correspond to (100), (020) and (110) crystal planes of α phase. The diffraction peak at 2*θ* = 20.7° that corresponds to (110) and (200) reflections of orthorhombic β phase appeared in VM spectra. The diffraction peak of β phase became evident with the increase in PMMA content, also indicating that PMMA was beneficial to the formation of β phase. And, the disappears of the peak at 17.8° represents the reduction of α crystal structure. The possible reason is that with the increase of PMMA content, more β phase was induced, while the content of α phase was relatively reduced. Furthermore, diffraction peaks in the spectra of preheating samples became sharp, suggesting the variations of crystallinity that was calculated from the following equation based on XRD patterns.

(2) $$\mathrm{crystallinity}\left(X_{c}\right)=\;\frac{S_{c}}{S_{c}+S_{a}}\;\times 100\%$$

where, *S_c_* and *S_a_* represent the sum of areas of crystalline parts and amorphous parts in XRD diffraction peaks, respectively. Peak separation and area calculation were all done through JADE. As shown in Figure 4f, *X_c_* of the casting films was increased after preheating procedure that prolonged the crystallization time. Due to volume effect, the increase of PMMA content decreased the *X_c_* of VM. 

### 3.2. β Phase Fractions and Crystallinities of PVDF during Biaxial Stretching

As mentioned above, mechanical stretching is seen as a simple and efficient method to increase the fraction of β phase. In the present work, the biaxial stretching device composed of 28 fixtures (Figure 1b) that should create a complex mechanical field and affect stress in different regions. The directions of stress are different along machine direction (MD), transverse direction (TD), and diagonal direction (DD). More importantly, stress value may also vary in different regions, including center (c), edges (d2, p2), and mediate regions (d1, p1).

**Figure 5:**
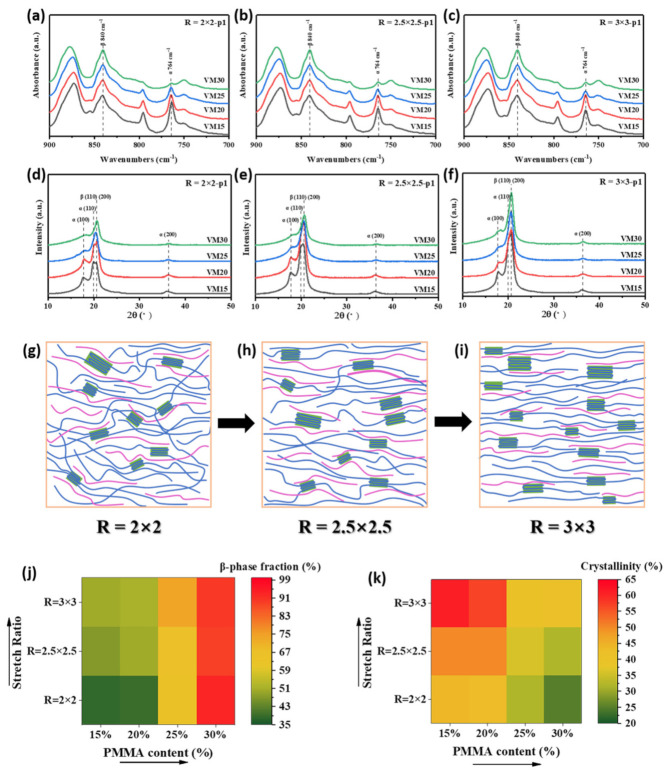
(**a**–**c**) FTIR spectra and (**d**–**f**) XRD patterns for the location of p1 in stretching VM films with ratios of 2 × 2, 2.5 × 2.5 and 3 × 3; (**g**–**i**) Schemes of crystal orientation in the film; (**j**) β phase fractions and (**k**) crystallinity versus PMMA content and stretching ratio.

For convenience, we investigated the β phase fraction and crystallinity of PVDF on p1 location to clarify the effect of mass ratio and stretching ratio. FTIR spectra for p1 location of biaxial stretching films are shown in Figure 5a–c. It can be seen from equation 1 that the β phase fraction depends on the peak of β phase at 840 cm^−1^ and the peak of α phase at 764 cm^−1^. To compare the β phase changes in different VM films with stretching ratios of 2 × 2, 2.5 × 2.5 and 3 × 3, characteristic peaks were normalized at β phase peak of 840 cm^−1^. Peaks at 764 cm^−1^ assigning to α phase showed a clear trend, i.e., the peak was weakened by increasing PMMA content. 

As shown in Figure 5d–f, the β phase diffraction peak at 2θ = 20.7° that corresponds to the (110) and (200) crystal planes appeared, and became obvious with the increase in PMMA content and stretching ratio. Schemes of crystals and polymer chains orientation in thin films are shown in Figure 5g–i. The orientation became increasingly obvious when the stretching ratio was increased to *R* = 3 × 3. It is already known that stretching at a high strain rate can result in efficient α-β evolution. The lamellae are aligned in the stretching direction and enough critical stress was introduced to make the rotation around C–C bonds possible [36].

The β phase fraction and crystallinity were also calculated. As shown in Figure 5j, the increase of PMMA content and stretching ratio enhanced the β phase fraction. At the PMMA content of 15 wt% and the stretching ratio of 2 × 2, the β phase fraction was as low as 38%. When the stretching ratio was increased to 2.5 × 2.5 and 3 × 3, the average β phase fractions were increased to 47% and 51%, respectively, which are higher than the initial preheated VM15 (37%). The excessive stretching ratio facilitated the evolution of α phase to β phase [36].

Similarly, at a constant stretching ratio of 3 × 3, average β phase fractions were 55%, 71% and 89% when the PMMA contents were 20 wt%, 25 wt%, and 30 wt%, respectively. There was still an increasing trend compared with the case before stretching. However, the effect of biaxial stretching on the amount of β phase crystals was not obvious when PMMA content reached 30%. The interaction between the CH_2_ groups in PVDF and the carbonyl (C=O) groups in PMMA serves as the key role to induce β phase. We argue that less carbonyl groups might exist in the interface between PVDF and PMMA domains when high amount of PMMA to some extent phase-separated from PVDF matrix.

The crystallinity of PVDF as shown in Figure 5k was increased by biaxial stretching. At the PMMA content of 15 wt%, the average crystallinity was 45%, 50% and 62%, corresponding to the stretching ratios of 2 × 2, 2.5 × 2.5 and 3 × 3, respectively, which is obviously higher than the initial preheating VM15 (34%). Stretching can facilitate the orientations of macromolecular chains and the formation of crystals (Figure 5g–i). Similarly, the crystallinity was suppressed by the addition of PMMA. At the stretching ratio of 3 × 3, the average crystallinity was 57%, 43% and 40% corresponding to PMMA content of 20, 25 and 30 wt%, respectively, which is still higher than the corresponding initial preheating samples (31%, 29%, and 26% as shown in Figure 4d). 

In comparison with the reported methods, the comparably high β phase fraction of 93% was achieved by biaxial stretching at the PMMA content of 30 wt% and the stretching ratio of 2 × 2. And according to the previous results, the β phase fraction can be well controlled by changing the PMMA content and stretching ratio. 

**Figure 6:**
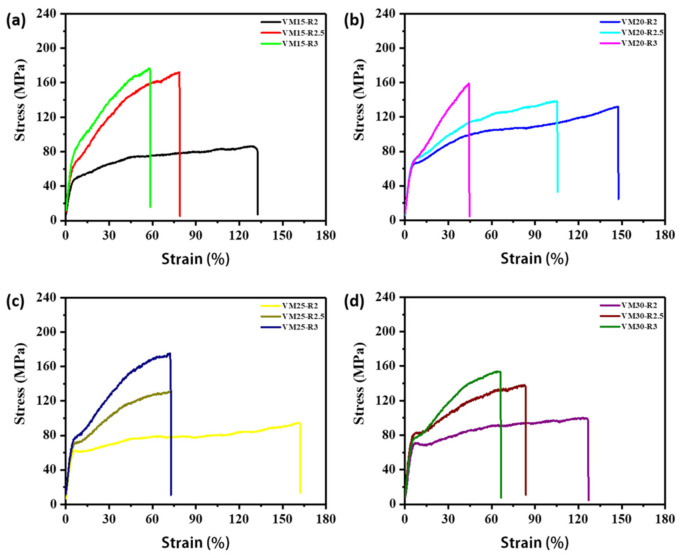
The stress-strain curves of biaxial stretching PVDF/PMMA blend films with varied compositions, (**a**) VM15, (**b**) VM20, (**c**) VM25 and (**d**) VM30.

The tensile analysis of biaxial stretching VM films with varied compositions were performed. Figure 6 shows the stress-strain curves of VM films on the location close to p1. The tensile direction was along MD. Due to the orientation of polymer molecular chains and the orientation induced crystallization in PVDF, the stress-strain curves showed apparent strain hardening phenomenon. The mechanical properties are listed in Table 3. When the stretching ratio was increased from 2 × 2 to 3 × 3, the elongation at break decreased apparently because the flexibility of polymer chains was reduced after biaxial stretching. Tensile strength and Young′s modulus were increased, which is attributed to the orientation of molecular chains along the stretching direction. There is no obvious trend in mechanical performance between PVDF/PMMA blends with varied compositions (from VM15 to VM30) possible due to the different mechanical properties of PMMA and PVDF.

### 3.3. Distribution of β Phase Fractions and Crystallinities of PVDF on Biaxially Stretched Films

**Figure 7:**
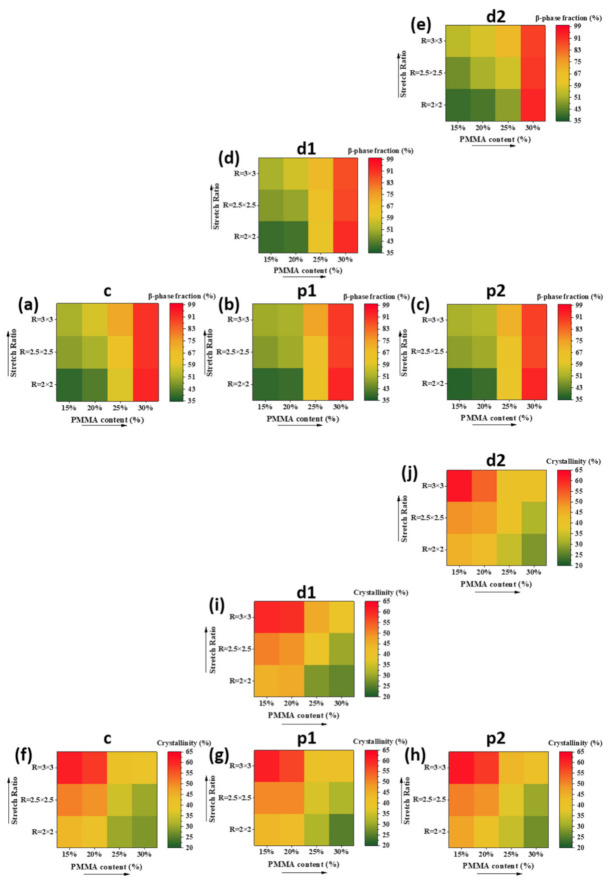
β phase fractions versus PMMA content and stretching ratio for the directions of (**a**) c, (**b**) p1, (**c**) p2, (**d**) d1 and (**e**) d2. And crystallinities versus PMMA content and stretching ratio for the locations of (**f**) c, (**g**) p1, (**h**) p2, (**i**) d1 and (**j**) d2.

Finally, we studied the distributions of β phase fractions and crystallinities of PVDF on biaxially stretched films. The original FTIR curves and XRD patterns are shown in supporting information. Here, the calculated β phase fractions and crystallinity of films on the other four locations (c, p2, d1, and d2) are shown in Figure 7 and compared with those on location p1. 

It was found that on each location, the effects of mass ratio and stretching ratio on β phase fractions and crystallinities of PVDF were similar, i.e., on one hand, the increase in PMMA content increased the β phase fractions but reduced the crystallinity, and on the other hand, it is stretching ratio that increased both the β phase fractions and crystallinity of PVDF.

More importantly, as shown in Figure 7, the difference of β phase fractions on different locations were neglected, indicating good uniformity of the distribution of β phase on the films. More specifically, the mean absolute deviation of β phase fractions on different locations was between 0.3% and 4.3%. In addition, the distributions of crystallinity were uniform as well on different locations of the films. The result shows that whenever a large biaxially stretched film is prepared, each small region has a similar β phase fraction and crystallinity of PVDF, which is important for the property stability. 

It is necessary to investigate the variations of stress during biaxial stretching to understanding how a complex shearing field affects the evolution of α-β phase transition. Finite element method (FEM) simulation was practiced through COMSOL.

As shown in Figure 1b, 28 fixtures (10.8 mm × 10.8 mm^2^) were used to clamp the film with the initial length × width of 10 cm × 10 cm^2^. Here, Yeoh hyperelastic material model was used to simulate the stretching stage of PVDF [45,46]. The strain energy density (*W*) can be expressed by the following equation:

(3) $$W=W_{iso}+W_{vol}=\overset{3}{\underset{p=1}{\sum }}C_{p}{\left(\bar{I_{1}}-3\right)}^{p}+\overset{3}{\underset{k=1}{\sum }}D_{k}{\left(J-1\right)}^{2k}$$

where, *W_iso_* is equal volume strain energy density, *W_vol_* is volumetric strain energy density, $\bar{I_{1}}$ is invariant of equal-body right Cauchy-Green tensor, *C_p_* and *D_k_* are material parameters. The parameters *D_k_* is not considered by assuming that the material is incompressible (*J* = 1). 

The second Piola-Kirchhoff stress tensor *S_a_* can be expressed:

(4) $$S_{a}=\frac{-V_{s}J}{R_{a}^{2}}+2\left(J^{-\frac{2}{3}}\left(\frac{\partial W_{iso}}{\partial \bar{I_{1}}}+\bar{I_{1}}\frac{\partial W_{iso}}{\partial \bar{I_{2}}}\right)-J^{-\frac{4}{3}}\frac{\partial W_{iso}}{\partial \bar{I_{2}}}R_{a}^{2}-\frac{1}{R_{a}^{2}}\left(\frac{\bar{I_{1}}}{3}\frac{\partial W_{iso}}{\partial \bar{I_{1}}}+\frac{2\bar{I_{2}}}{3}\frac{\partial W_{iso}}{\partial \bar{I_{2}}}\right)\right)$$

where, *R_a_* = *L*/*L*_0_ is the principal stretching ratio, and *L* represents the deformation length of a sample, *L*_0_ represents original length. *V_s_* is volume stress, and $\bar{I_{2}}$ is also the invariant of equal-body right Cauchy-Green tensor. The strain invariants, $\bar{I_{1}}$ and $\bar{I_{2}}$, are functions of the principal stretching ratios *R_a_* in the ath principal direction (*a* = 1, 2 and 3):

(5) $$\bar{I_{1}}=R_{1}^{2}+R_{2}^{2}+R_{3}^{2}$$

(6) $$\bar{I_{2}}=\frac{1}{R_{1}^{2}}+\frac{1}{R_{2}^{2}}+\frac{1}{R_{3}^{2}}$$

Assuming the material is incompressible (*J* = 1), the stretching ratio *R_a_* of biaxial deformation in the isotropic hyperelastic material is [47]:

(7) $$R_{1}=R_{2}=R,\;R_{3}=R^{-2}$$

During biaxial stretching, *S*_3_ = 0, *S*_1_ and *S*_2_ can be expressed as the following equation by eliminating volume stress *V_s_*.

(8) $$S_{1}=S_{2}=2\left(1-\frac{1}{R^{6}}\right)\left(\frac{\partial W_{iso}}{\partial \bar{I_{1}}}+R^{2}\frac{\partial W_{iso}}{\partial \bar{I_{2}}}\right)$$

Finally, the first Piola-Kirchhoff stress tensor *P* achieved through *P* = *RS*_1_ is expressed by the equation:

(9) $$P=2\left(R-\;R^{-5}\right)\overset{3}{\underset{n=1}{\sum }}nC_{n}{\left(2R^{2}+\frac{1}{R^{4}}-3\right)}^{n-1}$$

where, *C_n_* (*n* = 1, 2, 3) are Yeoh material parameters used in simulation. The stress-strain curves of PVDF at different temperatures have been studied in a previous research [48]. The stress (*P*) and strain ((*R* − 1) × 100%) were put into equation (9) to calculate *C*_1_, *C*_2_, and *C*_3_ as shown in Figure 7a. It can be found that the simulated curve agreed well with the stress-strain behavior of PVDF. In addition, according to a previous report [49] and our results, the introduction of PMMA did not affect the stress-strain behaviors of VM. Therefore, Yeoh hyperelastic material model and the same *C*_1_, *C*_2_, and *C*_3_ were used to simulate the relative stress and strain values. 

**Figure 8:**
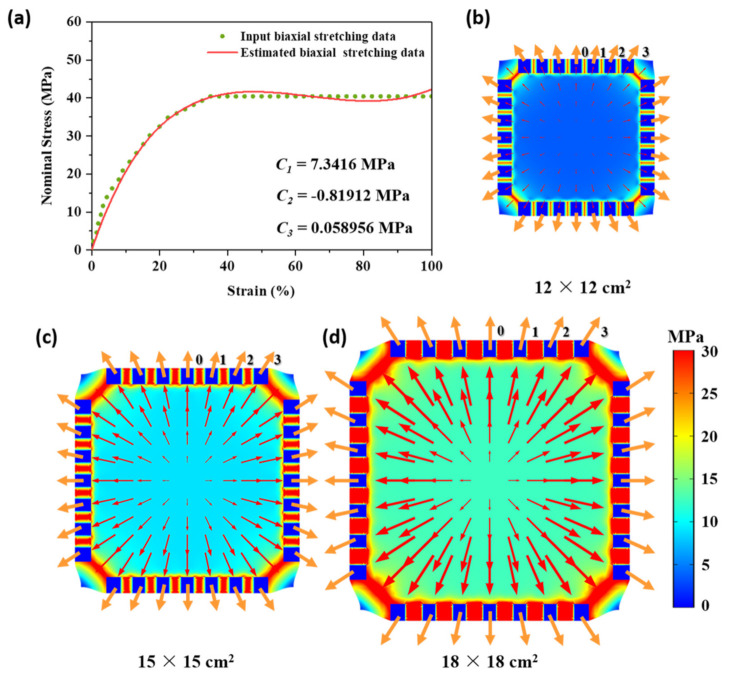
(**a**) The stress-strain curves of input biaxial stretching date and the estimated biaxial stretching date. (**b**,**c**) COMSOL simulation results for the stretching size of (**b**) 12 × 12 cm^2^, (**c**) 15 × 15 cm^2^ and (**d**) 18 × 18 cm^2^.

To achieve the uniform stretching along both MD and TD, the stretching velocity and direction of each clamp were different. As shown in Figure 1a, *V*_0_, *V*_1_, *V*_2_, and *V*_3_ were set as 1, 1.02, 1.09, and 1.19 mm/s, respectively, and the angles between *V*_1_, *V*_2_, *V*_3_ and *V*_0_ were 11.25°, 22.5°, and 33.75°. The film was stretched to the final length × width of 12 cm × 12 cm, 15 cm × 15 cm, and 18 cm × 18 cm. Figure 8b–d show the stress direction (indicated by red arrows) and values (indicated by color from blue to red, shown in legend in Figure 8d) on the film during stretching. The stress directions were distributed outwards uniformly. The directions of the simulated stress vectors (1, 2, 3 in Figure 8d) that were averagely distributed between MD and DD were the same as the moving directions of the three clamps. More importantly, the stress values were almost the same all over the film no matter which stretching ratio was used. The increase in stretching ratio from 1.2 to 1.5 and 1.8 increased the stress to 26 MPa. 

The effect of tensile stress has been studied before. Stress plays a key role in the transformation from α phase to β phase by leading to the orientation of the polymer chain [37,38,40,41,50,51]. According to the simulation results on stress distribution and value, we argue that the uniform stress distribution should cause the same orientation level on different locations. Besides, the increase in stress with stretching ratio resulted in the increase in orientation as well, leading to the change of β phase fractions and crystallinities of PVDF in PVDF/PMMA blends.

## 4. Conclusions

When the casting films that went through both extrusion and casting stages were concerned, β phase was observed compared with the melt samples without mechanical field. The β phase fraction of the casting films, however, was decreased after preheating. 

In following biaxial stretching, there was still an increasing trend of β phase fraction compared with the cases before stretching due to mechanical field. The increase in PMMA content increased the β phase fraction but reduced the crystallinity. It is stretching ratio that increased both the β phase fraction and crystallinity of PVDF. The highest β phase fraction of 93% was achieved at the PMMA content of 30 wt% and the stretching ratio of 2 × 2. The highest crystallinity degree of 62% was achieved at the PMMA content of 15 wt% and the stretching ratio of 3 × 3. The biaxial stretch process also significantly improved the mechanical properties of the film.

The distributions of β phase and crystallinity were uniform on different locations of the films possibly due to the uniform stress distribution all over the film with the aid of Finite element method simulation, leading to similar chain orientation levels. 

This work shows that biaxially stretching can be a candidate method to create PVDF/PMMA films with uniform distributions of comparable β phase and crystallinity degree of PVDF.

## Figures and Tables

**Table 1:** List of PVDF/PMMA blend (VM) films.

Code	Material Contents wt%		Stretching Ratio (*R*)
	PVDF	PMMA	
VM15	85	15	2 × 2, 2.5 × 2.5, or 3 × 3
VM20	80	20	
VM25	75	25	
VM30	70	30	

**Table 2:** The thickness of biaxial stretch films.

Stretch Films	Stretching Ratio R	Thickness (μm)
VM15	2 × 2	47
	2.5 × 2.5	26
	3 × 3	22
VM20	2 × 2	54
	2.5 × 2.5	41
	3 × 3	30
VM25	2 × 2	49
	2.5 × 2.5	32
	3 × 3	23
VM30	2 × 2	43
	2.5 × 2.5	34
	3 × 3	31

**Table 3:** Mechanical properties of PVDF/PMMA blends with varied compositions.

Code	Elongation at Break (%)	Tensile Strength (MPa)	Young’s Modulus (MPa)
VM15-R2	132	86	4200
VM15-R2.5	79	172	6492
VM15-R3	58	176	7013
VM20-R2	147	131	4530
VM20-R2.5	105	138	4945
VM20-R3	44	159	4990
VM25-R2	162	94	4139
VM25-R2.5	73	131	6269
VM25-R3	72	175	6740
VM30-R2	127	100	3113
VM30-R2.5	83	138	3837
VM30-R3	66	154	4636

## Data Availability

The data presented in this study are available in this article.

## Supplementary Materials

The following are available online at https://www.mdpi.com/2073-4360/13/7/998/s1, Figure S1: The dimensions of (a) the die, (b) cooling roll and the rotation speed, Figure S2: FTIR spectra of biaxial stretching PVDF/PMMA blend films with varied compositions (from VM15 to VM30) and stretch ratios at the locations of (a) c, (b) p1, (c) p2, (d) d1 and (e) d2. The results for stretching ratios of 2 × 2, 2.5 × 2.5, 3 × 3 are shown in (x − 1), (x − 2), and (x − 3), Figure S3: XRD patterns of biaxial stretching PVDF/PMMA blend films with varied compositions (from VM15 to VM30) and stretch ratios at the locations of (f) c, (g) p1, (h) p2, (i) d1 and (j) d2. The results for stretching ratios of 2 × 2, 2.5 × 2.5, 3 × 3 are shown in (x − 1), (x − 2), and (x − 3), Table S1: Material parameters of PVDF and PMMA.
