# Supplementary material for: Crystallinity and β Phase Fraction of PVDF in Biaxially Stretched PVDF/PMMA Films
Source: Polymers (Basel). 2021 Mar 24;13(7):998. doi: 10.3390/polym13070998 (PMC8037939; doi:10.3390/polym13070998)
Supplement: Supplementary file 1 [file polymers-13-00998-s001.pdf]

Supporting Information

Crystallinity and β phase fraction of PVDF in biaxially stretched PVDF/PMMA films

Ye Zhou[a,b,c], Wenting Liu[a,b,c], Bin Tan[d], Cheng Zhu[a,b,c,#], Yaru Ni[a,b,c,#], Liang Fang[a,b,c,#], Chunhua Lu[a,b,c], Zhongzi Xu[a,b,c]

[a] State Key Laboratory of Materials-Oriented Chemical Engineering, College of Materials Science and Engineering, Nanjing Tech University, Nanjing 210009, P. R. China.

[b] Jiangsu Collaborative Innovation Center for Advanced Inorganic Function Composites, Nanjing Tech University, Nanjing 210009, P. R. China.

[c] Jiangsu National Synergetic Innovation Center for Advanced Materials (SICAM), Nanjing Tech University, Nanjing 210009, PR China.

[d] Department of Chemical Engineering and Materials Science, Michigan State University, East Lansing, MI 48824, USA.

[#] Corresponding authors:

Zhucheng415@163.com (C. Zhu)

nyr@njtech.edu.cn (Y. Ni)

Lfang@njtech.edu.cn (L. Fang)

Table S1. Material parameters of PVDF and PMMA.

Material	Molecular weight	Chemical formula	Density g/cm^3	Melt flow g/10min
PVDF	310 k	$-(C_2H_2F_2)_n-$	1.78	20.9
PMMA	120 k	$-[CH_2C(CH_3)(COOCH_3)]_n-$	1.2	16

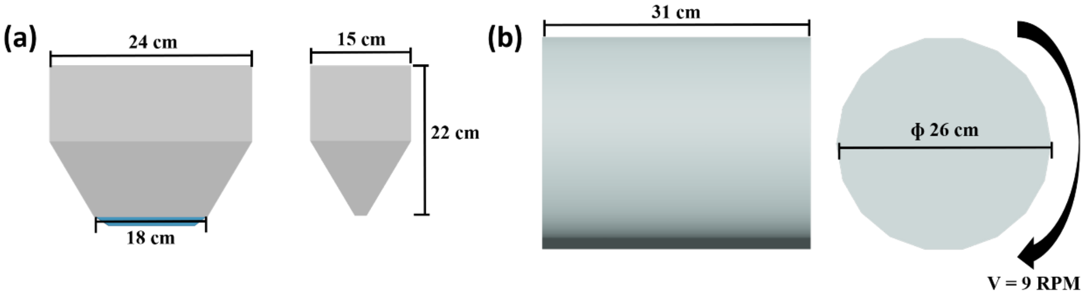

Figure S1. The dimensions of (a) the die, (b) cooling roll and the rotation speed.

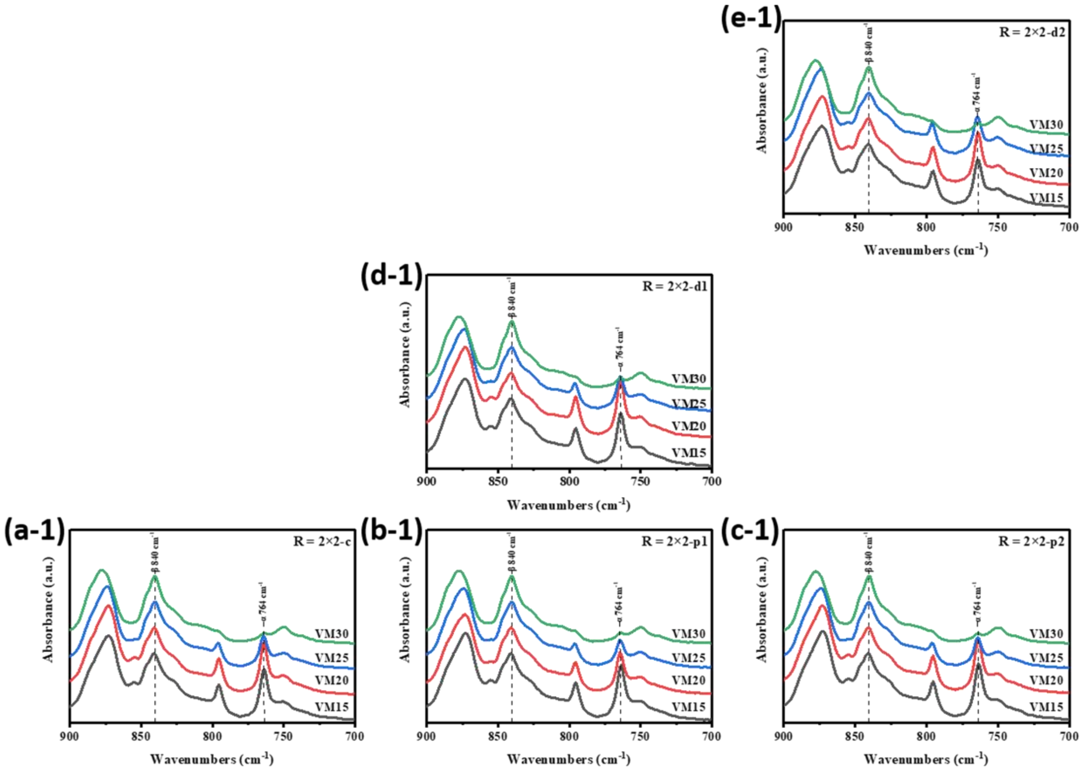

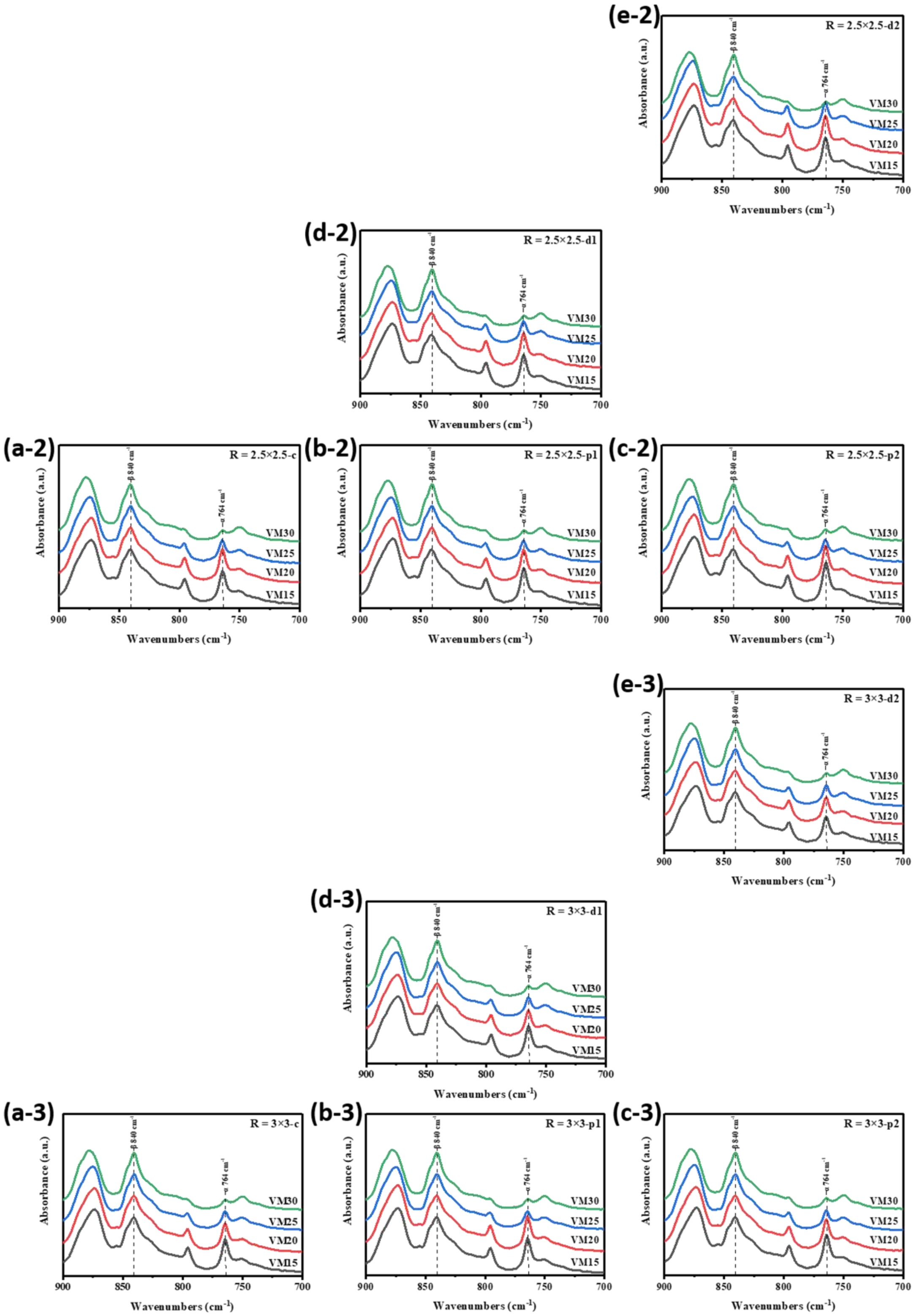

Figure S2. FTIR spectra of biaxial stretching PVDF/PMMA blend films with varied compositions (from VM15 to VM30) and stretch ratios at the locations of (a) c, (b) p1, (c) p2, (d) d1 and (e) d2. The results for stretching ratios of 2×2, 2.5×2.5, 3×3 are shown in (x-1), (x-2), and (x-3).

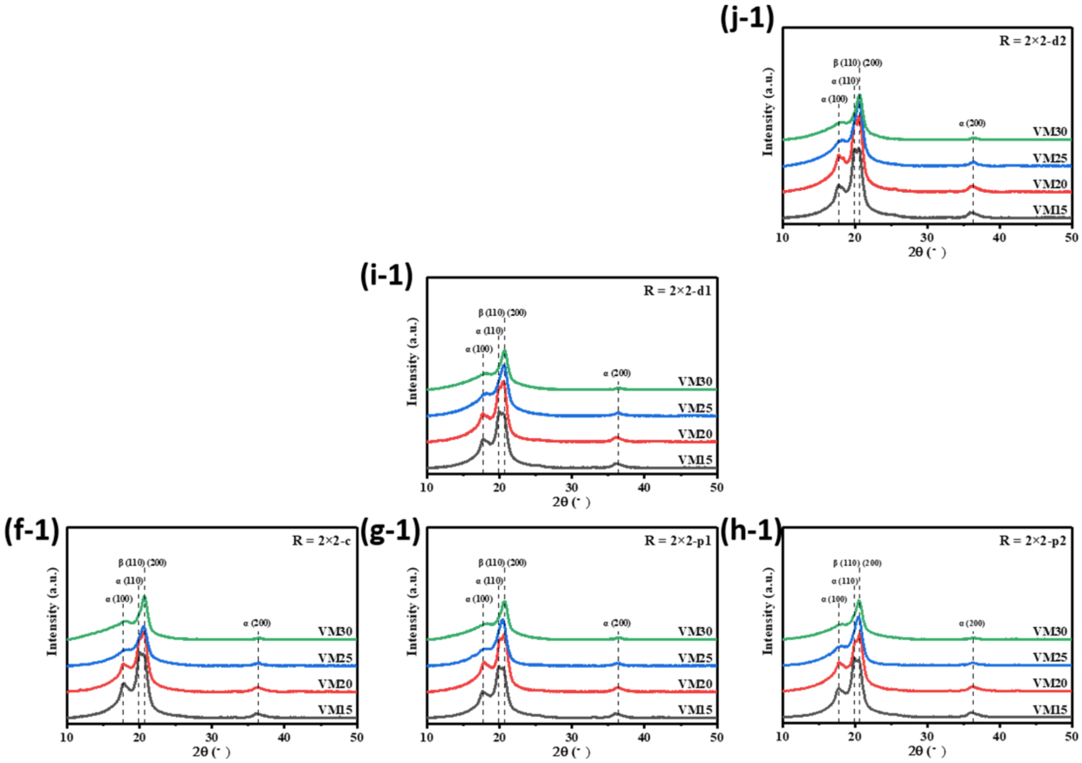

(j-1)
R = 2×2-d2
(i-1)
R = 2×2-d1
(f-1)
R = 2×2-c
(g-1)
R = 2×2-p1
(h-1)
R = 2×2-p2
Intensity (a.u.)
2θ (°)
β (110) (200)
α (110)
α (100)
α (200)
VM30
VM25
VM20
VM15
10
20
30
40
50

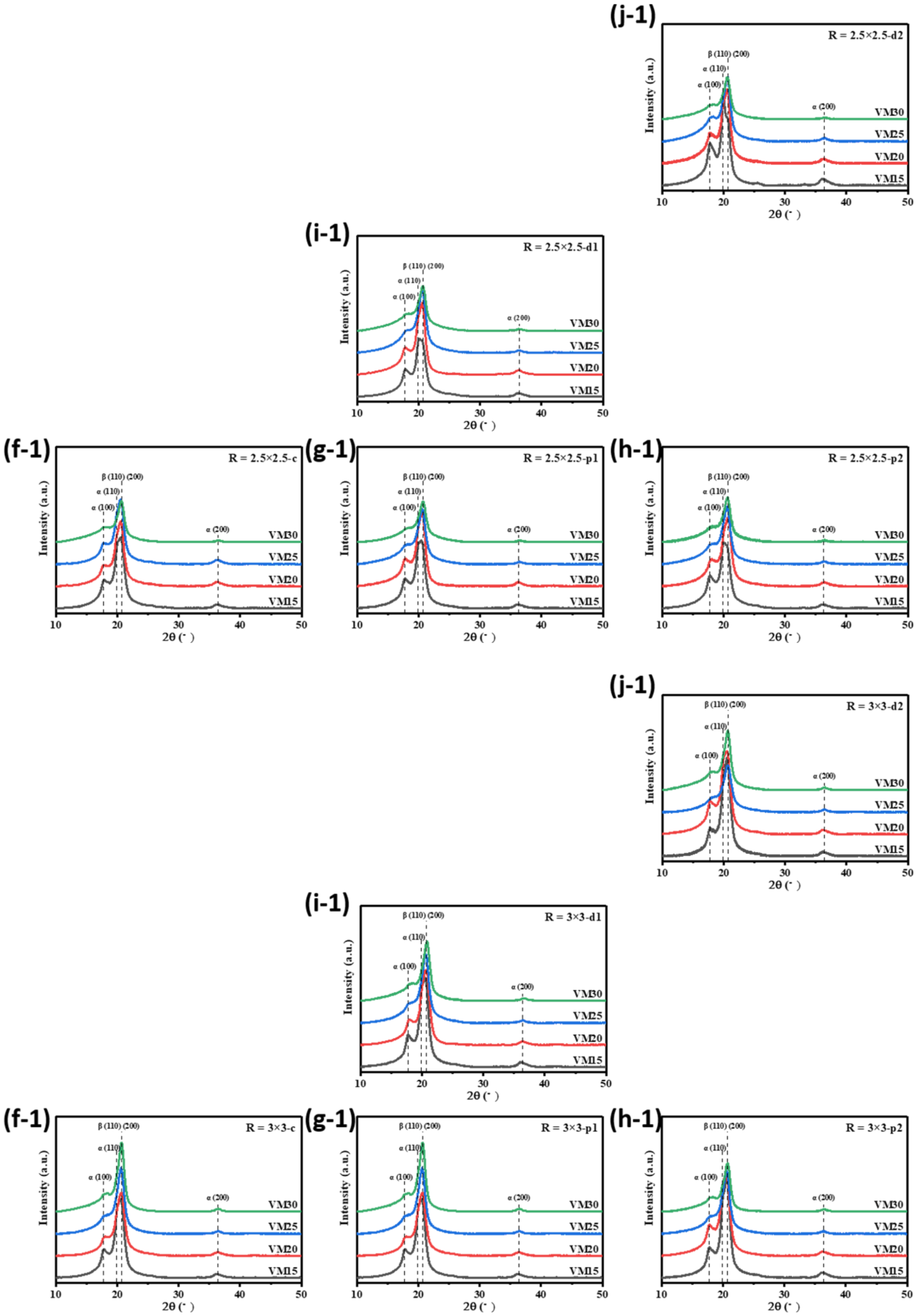

Figure S3. XRD patterns of biaxial stretching PVDF/PMMA blend films with varied compositions (from VM15 to VM30) and stretch ratios at the locations of (f) c, (g) p1, (h) p2, (i) d1 and (j) d2. The results for stretching ratios of 2×2, 2.5×2.5, 3×3 are shown in (x-1), (x-2), and (x-3).
